# Interaction between β-hexachlorocyclohexane and *ADIPOQ* genotypes contributes to the risk of type 2 diabetes mellitus in East Chinese adults

**DOI:** 10.1038/srep37769

**Published:** 2016-11-24

**Authors:** Shushu Li, Xichen Wang, Lu Yang, Shen Yao, Ruyang Zhang, Xue Xiao, Zhan Zhang, Li Wang, Qiujin Xu, Shou-Lin Wang

**Affiliations:** 1 Key Lab of Modern Toxicology of Ministry of Education, School of Public Health, Nanjing Medical University, 101 Longmian Avenue, Nanjing 211166, P. R. China; 2 State Key Lab of Reproductive Medicine, Institute of Toxicology, Nanjing Medical University, 101 Longmian Avenue, Nanjing 211166, P. R. China; 3 Lake Research Center, Chinese Research Academy of Environmental Sciences, 8 Dayangfang, Anwai Beiyuan, Beijing 100012, P. R. China

## Abstract

Growing evidence links environmental exposure to hexachlorocyclohexanes (HCHs) to the risk of type 2 diabetes mellitus (T2DM), and *ADIPOQ* that encodes adiponectin is considered as an important gene for T2DM. However, the role of *ADIPOQ*-HCH interaction on T2DM risk remains unclear. Thus, a paired case-control study was conducted in an East Chinese community. A total of 1446 subjects, including 723 cases and 723 controls matched on age, gender and residence, were enrolled, and 4 types of HCH isomers were measured in serum samples using GC-MS/MS. Additionally, 4 candidate *ADIPOQ* SNPs (rs182052, rs266729, rs6810075, and rs16861194) were genotyped by TaqMan assay, and plasma adiponectin was measured using ELISA. No associations between 4 SNPs and T2DM risk were found, but T2DM risk significantly increased with serum levels of β-HCH (*P* < 0.001). Furthermore, the synergistic interaction between β-HCH and rs182052 significantly increased T2DM risk (OR _*I-additive model*_ = 2.20, OR _*I-recessive model*_ = 2.13). Additionally, individuals carrying only rs182052 (A allele) with high levels of β-HCH had significant reduction in adiponectin levels (*P* = 0.016). These results indicate that the interaction between rs182052 and β-HCH might increase the risk of T2DM by jointly decreasing the adiponectin level and potentially trigger T2DM development.

Recently, the prevalence of diabetes has increased significantly and is reaching epidemic proportions. In China, the overall prevalence of type 2 diabetes mellitus (T2DM) is estimated to be 11.6% in adult populations^1^. T2DM is a chronic, complex disease, and mounting evidence suggests that T2DM may arise from the interaction of both genetic and environmental factors.

Genetic factors may predispose an individual to T2DM, as indicated by high family aggregation^2^. Recent genome-wide studies^3^,^4^,^5^ have mapped a susceptibility locus for T2DM to chromosome *3q27,* where the *ADIPOQ* gene that encodes adiponectin is located. Several studies^6^,^7^ have reported that plasma adiponectin levels are reduced in subjects with obesity, T2DM, insulin resistance and cardiovascular diseases. Compared to normal animals, adiponectin/ACRP30 knockout mice were found to be more sensitive to diet-induced insulin resistance^8^. In addition, data suggest that polymorphisms of *ADIPOQ* may be correlated with plasma adiponectin levels, obesity, insulin resistance and T2DM^9^,^10^. For example, rs266729 and rs16861194, located in the 5′ region proximal to the *ADIPOQ* gene, might affect adiponectin promoter activity in adipocytes and increase the risk of T2DM in Finnish as well as Han Chinese populations^11^.

Growing evidence has correlated the risk of T2DM with environmental exposure to persistent organic pollutants (POPs)^12^,^13^, including hexachlorocyclohexanes (HCHs)^14^. HCHs, most widely used as pesticides, were produced commercially by the chlorination of benzene, including α-HCH, β-HCH, γ-HCH and δ-HCH isomers^15^, of which β-HCH has the longest half-life. Although HCHs were banned several decades ago in most countries due to possible harms to wildlife and humans^16^,^17^, they continue to be detected in the environment^18^, human serum and breast milk^19^,^20^. Several population studies have suggested that long-term exposure to HCHs may result in strong insulin resistance, dyslipidemia, obesity and T2DM^21^.

Currently, gene-environment interactions are widely considered to underlie the etiology of chronic non-communicable diseases, including diabetes^22^. In general, the impact of gene-lifestyle interactions on T2DM have been more frequently reported. Previous studies^23^,^24^ have emphasized the importance of the conventional lifestyle factors, such as physical activity, body mass index (BMI), physical and mental stress, dietary habits, and smoking, rather than prior exposure to environmental chemicals. However, researchers have increasingly focused on the effects of the interactions between genes and environmental pollutants on disease. Polymorphisms of xenobiotic metabolizing genes (*CYP17A1* or *GST*) and environmental exposure to HCHs have been reported to contribute to the risk of male infertility and fetal growth restriction^25^,^26^,^27^. The interaction between genetic polymorphisms of *Cytochrome P450 (CYP), Glutathione S-transferase* (*GST*) *or GSTM1* and serum β-HCH levels might magnify the risk of some cancers^28^,^29^,^30^. Research in Sweden suggests that interactions between polymorphisms in *AHR* (*R554K*) and AHR repressors (*AHRR P185A*) and serum levels of POP markers (p, p′-DDE and PCB153) may affect human semen quality^31^. However, few studies have studied the interactions between genes, environmental xenobiotics and the risk of T2DM.

In the present study, we first investigated the impact of the interaction between HCHs, and in particular β-HCH, and the *ADIPOQ* gene on the risk of T2DM. We conducted a paired case-control study, including 723 T2DM cases and 723 healthy controls, in an East Chinese population. Then, we examined the combined effect of β-HCH and the *ADIPOQ* gene on plasma concentrations of adiponectin to validate the interaction effects. This study attempts to reveal the effects of the synergistic nature of genetics and environmental factors on T2DM and help to provide a better understanding of the etiology of and preventive strategies for T2DM.

## Results

### Risk factors of T2DM in the study population

Study participants (723 diabetes and 723 controls matched on age, sex, and residence) included 482 men and 964 women (241 men and 482 women in each group); most of them were farmers (90.7%) with junior school education (92.7%). As shown in Table S1, the distribution of cigarette smoking and alcohol drinking was similar between cases and controls, but a family history of diabetes was reported more frequently in the case group than in control group (5.9% *vs* 2.2%). Cases were more likely to report hypercholesterolemia, hypertriglyceridemia, dyslipidemia, hypertension, obesity, low-HDLC, and high-LDLC than controls (*P* < 0.001). The conditional logistic regression analysis suggested that family history of diabetes, hypertension, dyslipidemia and obesity were independent risk factors for diabetes (Table S2); these variables were subsequently used as the covariates in the association between *ADIPOQ* genotypes and/or HCHs and the risk of T2DM.

### Association of ADIPOQ genotypes with the risk of T2DM

All the four SNPs (rs182052, rs266729, rs6810075, and rs16861194) were in Hardy–Weinberg equilibrium (*P* > 0.05), and the MAF of SNPs in the case and control groups were similar to that of HAPMAP-CHB (Supplemental Material, Table S5). As shown in Table 1, no significant association between the single *ADIPOQ* genotype and the risk of T2DM was observed in comparison with the corresponding wild-type and in either dominant or additive models.

### Association of the serum levels of HCHs with the risk of T2DM

As shown in Table S6, β-HCH had much a higher detection rate and level than the other HCH isomers in both cases and controls. Further, both the detection rate (71.2%) and geometric mean (0.575 ng/mL) of β-HCH were significant higher in cases than in controls (*P* < 0.001). To investigate the relationship between HCHs and the risk of T2DM, the total HCHs was trisected into three groups, as indicated by the distribution of concentrations in the controls, and the lowest tertile was used as a reference category. As shown in Table 2, the risk of T2DM increased with HCH concentration (*P*_−*trend*_ < 0.001), and this association was significant in the highest tertile group (OR: 2.31, 95% CI: 1.55–3.44). To identify the association between the risk of T2DM and concentrations of single HCH isomers, the participants were classified into three groups following the distribution of concentrations of HCH isomers in controls; in this classification scheme, concentrations below the limit of detection (LOD) were regarded as the reference group, and those with detectable values were categorized into the second and third medians with the median concentration as the cutoff point. As shown in Table 2, after adjusting for confounders, the concentration of β-HCH was significantly and positively associated with the risk of T2DM. The adjusted odds ratios (ORs) were 1.53 (95% CI: 1.09–2.06) and 2.56 (95% CI: 1.67–3.89) in Median 1 and 2 groups, respectively (*P*_−*trend*_ = 0.000). However, other HCH isomers showed no significant association with the risk of T2DM.

### Interaction between ADIPOQ genotypes and β-HCH on the risk of T2DM

The potential interaction between serum β-HCH levels and *ADIPOQ* genotypes on the risk of T2DM was evaluated using stratified *ADIPOQ* genotypes and the β-HCH levels of each subject considered as a continuous variable to be introduced into the conditional logistic regression model. As shown in Table 3, a statistically significant interaction was found between β-HCH and rs182052 (adjusted OR_*I- additive model*_ = 2.20, 95% CI: 1.39–3.49, *P*_*I- additive model*_ = 0.010; adjusted OR_*I- recessive model*_ = 2.13, 95% CI: 1.31–3.26, *P*_*I- recessive model*_ = 0.005), but the interaction between β-HCH and rs6810075or rs266729 was not significant (*P*_*I*_ > 0.0125). Unequivocally, the OR_*I*_ of the β-HCH and rs182052 interaction (OR_*I- additive model*_ = 2.20; OR_*I- recessive model*_ = 2.13) increased twice in comparison of either OR_β-HCH_ (OR_*I- additive model*_ = 1.15; OR_*I- recessive model*_ = 1.21) or OR_rs182052_ (OR_*I- additive model*_ = 0.86; OR_*I- recessive model*_ = 0.77), but the OR_*I*_ of β-HCH and other genotypes did not exceed the multiplicative terms of their main effects.

### Combined effects of rs182052 genotype and β-HCH on the adiponectin level

**Figure 1:**
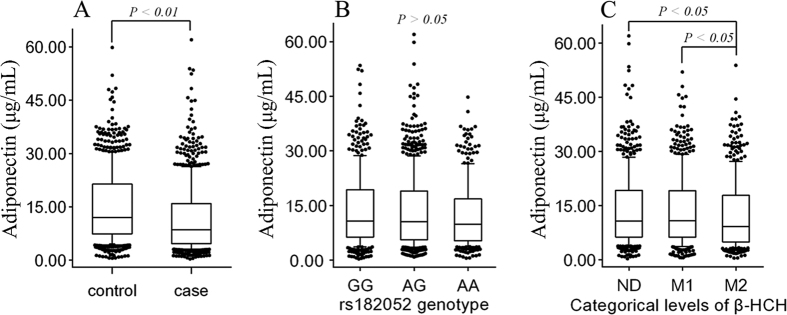
Plasma adiponectin levels among groups categorized by *rs182052* genotype or serum β-HCH levels. (**A**) The adiponectin levels in cases and controls. (**B**) The distribution of the adiponectin levels among *rs182052* genotype. (**C**) The distribution of the adiponectin levels among β-HCH groups. ND, not detectable; M1 and M2, median of detectable levels. Data were presented in Box (10–90 percentile) and whiskers, *p* values were calculated by nonparameteric test.

To verify the impact of interaction between β-HCH and rs182052 on the risk of T2DM, the plasma levels of adiponectin was measured. As shown in Fig. 1, the median level of adiponectin was significantly lower in cases (13.34 μg/mL) than in controls (8.61 μg/mL; *P* < 0.01; Fig. 1A). After stratification, participants with rs182052 genotypes (AA) had a declined adiponectin levels in comparison with those who carried wild-type *ADIPOQ*; this difference was not significant (GG) (*P* > 0.05, Fig. 1B). However, participants with high levels of β-HCH had lower adiponectin levels than lower β-HCH level groups (reference or Median 1 groups) (*P* < 0.05, Fig. 1C). Furthermore, the levels of adiponectin in the rs182052 genotype (AG/AA) group and high β-HCH exposure group were lower than the levels in the wild type (GG) and low β-HCH exposure groups, but these differences were not significant (Table 4). However, individuals carrying rs182052 (mutant allele A, AG/AA) with high levels of β-HCH had a significant reduction in adiponectin levels (8.04 μg/mL) in comparison of those with low levels of β-HCH (*P* = 0.016, Table 4).

## Discussion

In this study, we conducted a paired case-control study (723 cases and 723 controls) in an East Chinese populations and investigated impact of the interaction between 4 candidate SNPs of *ADIPOQ* and serum HCHs levels on the risk of T2DM. The results from the multivariate analysis suggested that serum level of β-HCH, rather than other HCH isomers or any candidate *ADIPOQ* SNPs, were significantly associated with the risk of T2DM. Notably, interactions between β-HCH and rs182052 demonstrated significantly increased risk of T2DM (OR = 2.20, 95% CI: 1.39–3.49), which was consistent with the combined effect of β-HCH and rs182052 on decreased levels of plasma adiponectin (8.04 μg/mL), a common predictor of diabetes.

Genome-wide studies^32^ have mapped a susceptibility locus *ADIPOQ* for T2DM. However, a contrary view^33^ observed that rs182052, rs266729, and rs16861194 had no association with T2DM in Han Chinese populations; similar results were observed with rs182052 and rs6810075 among Japanese women. Likewise, our study did not find the statistical association between the 4 candidate *ADIPOQ* genotypes (rs182052, rs266729, rs6810075 and rs16861194) and the risk of T2DM. It is well established that HCHs play important role in the development of T2DM^34^,^35^. Several population studies have suggested that long-term exposure to HCHs results in strong insulin resistance, dyslipidemia, obesity and T2DM^21^. The β-HCH had the longest half-life; it may be the most toxicological HCH isomer with the highest detection rate, which is evidenced by its estrogenic effects in mammalian cells, laboratory animals, and humans. In the present study, the gross serum levels of HCHs were associated with increased risk of T2DM, but this association remained significant only in the β-HCH isomer (Table 1). In addition, β-HCH had a much higher detection rate and approximately 20-fold serum levels relative to other HCH isomers in the study populations (Table S6).

T2DM is a chronic non-communicable disease thought to arise from both genetic and environmental factors, and gene-environment interactions are widely considered to underlie its etiology^22^. Frequently, the logistic regression model (LRM)^36^,^37^ has been used to estimate the statistical interaction between risk factors by adding a product term. In LRM, if the product term (γ, interaction term of rs182052-β-HCH in our study) is significant on a multiplicative scale, then interaction exists. In the present cLRM analysis (Table 3), a synergistic interaction on the risk of T2DM was did observed between rs182052 and β-HCH (Table 3), though *ADIPOQ* genotypes or β-HCH alone still similarly increased the risk of T2DM (Tables 1 and 2). The OR_*I*_ for the interaction between rs182052 and β-HCH in additive or recessive model was higher than that of either rs182052 or β-HCH alone; however, the OR_I_ in β-HCH and other genotypes was lower than that of β-HCH alone, indicating that the interaction between rs182052 and β-HCH might increase the risk of T2DM and that rs182052 modifies the effect of β-HCH on the risk of T2DM.

SNP rs182052 is located in intron 1 of *ADIPOQ* gene, and its minor allele A results in a loss of a Sp1-binding site and gain of a CCAAT/enhancer-binding protein (C/EBP) b-binding site, reducing adipocyte differentiation^38^,^39^. SNP rs182052 was reported to be significantly associated with lower plasma adiponectin^40^, a strong predictor of diabetes^41^, which was reflected in our finding that cases had lower adiponectin levels than controls (Fig. 1A). HCHs were linked to modulation of the alteration of adipokines, such as leptin, adiponectin, and resistin, in both *in vitro* and *in vivo* experiments^42^,^43^,^44^. Similar to the above results regarding the increased risk of T2DM, rs182052 showed a borderline, but β-HCH presented a significantly, declined level of adiponectin (Fig. 1B,C). Thus, it is likely that HCHs may induce inflammation and a decreased mitochondrial function in adipocytes, further decreasing adipocyte maturation, inhibiting adiponectin incretion, and subsequently leading to a risk of T2DM.

Furthermore, we evaluated the joint effects of rs182052 and β-HCH on the plasma adiponectin level. Only individuals carrying rs182052 (mutant allele A, AG/AA) with high levels of exposure to β-HCH had a significant reduction in the adiponectin level (8.04 μg/mL) when compared with those with low-level exposures to β-HCH (*P* = 0.016, Table 4) after stratified analysis, indicating that rs182052 and β-HCH jointly decrease plasma adiponectin level. Overall, the mutant A allele of rs182052 appeared to have no statistically significant effect on increasing the risk of T2DM; however, when combined with exposure to β-HCH, the interaction could trigger T2DM development. It seemed probably that rs182052 as a biological modifier might aggravate the reduction of adipocyte differentiation and maturation induced by exposure to HCHs. Researchers have found that DNA hypermethylation of the *ADIPOQ* promoter inhibits adiponectin transcription and mediates insulin resistance^45^. Additionally, abnormal adiponectin may lead to upregulation of hepatic IRS-2 via an IL-6 dependent pathway and enhanced insulin sensitivity^46^. Thus, it is more likely that exposure to β-HCH might mediate DNA methylation of *ADIPOQ* or lead to abnormal adiponectin levels and promote the development of T2DM, which is further modified by rs182052 via interaction.

There are several limitations to the present study. Owing to not a big sample size of case-control design, the interaction between β-HCH and rs182052 on T2DM risk needs to be further validated, and an independent enlarge population and/or a prospective cohort studies should be conducted. In addition, the biological mechanisms that how β-HCH–rs182052 interaction to affect adponectin and then aggravate the development of T2DM should be elucidated in future study.

To our knowledge, this is the first assessment of the impact of the interaction between *ADIPOQ* genotypes and β-HCH on the risk of T2DM in Chinese populations. Our findings suggest that the interaction between rs182052 and β-HCH might aggravate the risk of T2DM by jointly decreasing the adiponectin levels and triggering T2DM development. The gene-environment interaction model could help us understand this intrinsic black box in the pathophysiology of T2DM. Additional functional studies should be carried out to further elucidate the role of the interaction between *ADIPOQ* and β-HCH in the development of T2DM.

## Methods

### Study population

This paired case-control study was conducted independently of a cross-sectional investigation of a community population in the North Jiangsu province, located at East China, from May 2011 to August 2012. In total, 723 T2DM cases and 723 healthy controls matched on sex, age and residence were enrolled. Participants were considered as T2DM if they had been previously diagnosed in a hospital or had a fasting blood glucose ≥7 mmol/L validated at least twice in different periods and were subsequently diagnosed by a local hospital. Each subject was face-to-face interviewed by trained interviewers using a questionnaire to collect personal information, including demographic, family history of diseases, health status, and lifestyle data (Supplemental Materials). Then, two 5 mL venous blood samples were collected, and serum and plasma were separated immediately. The samples were then stored at −20 °C until the biochemical indices, adiponectin and HCH isomers, were determined. The study was approved by the Institutional Review Board of Nanjing Medical University, and all the procedures were in accordance with the prevailing ethical principles. All the participants signed informed consent prior to taking part in this study.

### Selection of ADIPOQ SNPs and genotyping assays

Based on NCBI database (http://www.ncbi.nlm.nih.gov/SNP), HapMap SNP database (http://www.hapmap.org), and Haploview software (Version 3.2), *ADIPOQ* SNPs meeting the following criteria were considered for inclusion: (1) common SNPs (MAF >5%) in Han Chinese populations; (2) SNPs previously reported as associated with other diseases, especially diabetes; (3) SNPs that have not been previously implicated in the pathogenic process of diabetes, especially in Han Chinese populations; and (4) SNPs with low linkage disequilibrium (LD) (r^2^ < 0.8). Thus, rs182052, rs266729, rs6810075 and rs16861194 of the *ADIPOQ* gene were selected for genotyping in the study.

Genomic DNA was obtained from white-blood cell fractions using the Qiagen Blood Kit (Qiagen) and following the manufacturer’s protocols. The 384-well ABI 7900HT Real-Time PCR System (Applied Biosystems, Foster City, CA, USA) was used for the TaqMan SNP Genotyping assay (Supplemental Material Fig. S1). The primer was designed and synthesized by GENEray Biotechnology (Shanghai, China), and sequences are listed in Supplemental Material, Table S3. To validate the results, 10% of the samples were randomly selected for repeated genotyping to assess the reproducibility, and the concordance rate was 100%.

### Measurement of serum HCH isomers

The single standard solutions of α-HCH, β-HCH, γ-HCH, and δ-HCH were purchased from Sigma-Aldrich Laboratories Inc. (St. Louis, MO, USA). All solvents, including methanol, hexane, dichloromethane, isopropyl alcohol, and methyltertbutylether (MTBE); the Classic Florisil SPE; and common supplies were purchased from ANPEL Laboratory Technologies Inc. (Shanghai, China).

Serum sample extraction, separation and cleanup were employed using a modification of methods described previously^47^, and HCHs were measured using GC-MS/MS; the specific details of the steps are described in the Supplemental Materials. The five-point calibration curves of HCHs standards (1 ng/mL, Fig. S2A) and a representative spectrum of serum sample (Fig. S2B) are shown in Supplemental Material, Fig. S2. Additionally, a more detailed description of the recovery rate, relative standard deviation (RSD), limit of detection (LOD), and range of levels in human serum are listed in Supplemental Material, Table S4. Preliminarily, β-HCH had a more than 60% higher detection rate than its congeners (α-HCH, γ-HCH and δ-HCH) in the screening of 250 randomly collected serum samples from the total population.

### Measurement of plasma adiponectin

Plasma concentrations of adiponectin were measured using a commercially available enzyme-linked immunosorbent assay kit (ELISA, CUSABIO, Wuhan, CHINA) and following the manufacturer’s instructions. Briefly, the diluted plasma sample (1:500) was put in 96-well plates (100 μL per well), and incubated at 37 °C for 2 hours before adding 100 μL of biotin-labeled antibody and incubating at 37 °C for 1 hour. After the sample was aspirated and washed with Wash Buffer (200 μL) three times, 100 μL HRP-avidin was added and incubated at 37 °C for 1 hour. Then, we aspirated and washed the well five times, added TMB Substrate (90 μL), incubated in the dark at 37 °C for 15–30 minutes, and stopped the reaction by adding 50 μL of Stop Solution. The optical density was determined at 450 nm within 5 minutes using a micro-plate reader (Infinite M200 Pro, Tecan Group, Switzerland). During the experiment, the adiponectin standard was synchronously tested with plasma samples to quantify the adiponectin content. The intra- and inter-assay coefficients of variation were 5.5–7.9% and 6.5%, respectively.

### Statistical analysis

#### General statistical analysis

All statistical analyses were performed in SPSS Version 17.0 (SPSS Inc., Chicago, USA) and STATA (Version 11). The χ^2^ test and Student’s t test for continuous variables were used to analyze differences in the distribution of demographics and genotypes between cases and controls. The Mann−Whitney U-test was used to analyze the difference in HCHs concentrations between groups. The Hardy–Weinberg equilibrium (HWE) for the distribution of each SNP was evaluated using goodness-of-fit χ^2^ tests to compare the observed genotype frequencies with expected ones among the controls. Odds ratios (ORs) and 95% confidence intervals (CI) were calculated using conditional logistic regression analysis. *P* < 0.05 was considered as statistical significance.

#### Statistical analysis for the interaction between β-HCH and ADIPOQ genotypes

The statistical analysis was performed in STATA (Version 11). Multiplicative interactions were tested using a conditional logistic regression model (cLRM) with covariate adjustment^48^ following the equation:


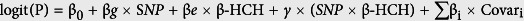


Logit(*P*) represents the natural logarithm of the ratio of the positive occurrence (case) probability to the negative occurrence (control) probability. *P* denotes the probability of having diabetes (case or control), the regression coefficients *β*_*g*_, *β*_*e*_ or 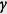 indicate the changes in logit(*P*) when the SNP, β-HCH, SNP × β-HCH or covar_i_ factor changes by one unit. SNP represents the *ADIPOQ* genotype coded in an additive genetic model (0 = wild homozygote, 1 = mutant heterozygote, 2 = mutant homozygote). The serum level of β-HCH was included in model as a continuous variable. *β*_*0*_ represents a constant; β_g_ and β_e_ are the main effects of SNP and β-HCH, respectively; and the product γ (SNP × β-HCH) is the interaction term. OR_e_ and OR_g_ represent the main effects of environment factor (OR_e_ = exp(*βe*)) and genetic factor (OR_g_ = exp (*βg*)), respectively. OR_I_ represents an interaction that exceeds the multiplicative term of the main effects of both the environment and genetic factors (OR_I_ = exp (γ) = OR_eg_/(OR_g_ × OR_e_)). Covar_i_ is the covariate to control for confounding factors, including family history of diabetes, hypertension, dyslipidemia, obesity and total lipids. The significant *P* for interaction was set as 0.0125 (0.05/4) using Bonferroni correction.

## Additional Information

**How to cite this article**: Li, S. *et al*. Interaction between β-hexachlorocyclohexane and *ADIPOQ* genotypes contributes to the risk of type 2 diabetes mellitus in East Chinese adults. *Sci. Rep.* **6**, 37769; doi: 10.1038/srep37769 (2016).

**Publisher’s note:** Springer Nature remains neutral with regard to jurisdictional claims in published maps and institutional affiliations.

## Supplementary Material

Supplementary Information

## Figures and Tables

**Table 1:** Association between *ADIPOQ* genotypes and the risk of T2DM.

Genotypes	Control No. (%)	Case No. (%)	OR (95% CI)^a^	*P*^b^
rs182052				
GG	215 (50.8)	208 (49.2)	1.00 (Reference)	—
AG	347 (49.1)	360 (50.9)	1.03 (0.76, 1.39)	0.875
AA	161 (50.9)	155 (49.1)	0.99 (0.67, 1.45)	0.943
AG/AA^c^	508 (49.7)	515 (50.3)	1.01 (0.76, 1.35)	0.927
GG/AG^d^	562 (59.7)	568 (50.3)	0.97 (0.70, 1.35)	0.862
rs266729				
CC	365 (49.4)	374 (50.6)	1.00 (Reference)	—
CG	282 (49.8)	284 (50.2)	0.95 (0.73, 1.26)	0.954
GG	76 (53.9)	65 (46.1)	0.76 (0.48, 1.21)	0.251
CG/GG^c^	358 (50.6)	349 (49.4)	0.91 (0.71, 1.18)	0.489
CC/CG^d^	647 (49.6)	658 (50.4)	0.77 (0.49, 1.22)	0.271
rs6810075				
TT	221 (52.4)	201 (47.6)	1.00 (Reference)	—
CT	350 (49.0)	365 (51.0)	1.13 (0.83, 1.53)	0.438
CC	152 (49.2)	157 (50.8)	1.17 (0.80, 1.72)	0.414
CT/CC^c^	502 (49.0)	522 (51.0)	1.14 (0.86, 1.52)	0.373
TT/CT^d^	571 (50.2)	566 (49.8)	1.09 (0.78, 1.52)	0.622
rs16861194				
AA	499 (51.1)	477 (48.9)	1.00 (Reference)	—
AG	198 (48.3)	212 (51.7)	1.10 (0.81, 1.48)	0.547
GG	26 (43.3)	34 (56.7)	1.38 (0.70, 2.73)	0.351
AG/GG^c^	224 (47.7)	246 (52.3)	1.13 (0.85, 1.50)	0.398
AA/AG^d^	697 (50.3)	689 (49.7)	1.35 (0.69, 2.65)	0.384

^a^Adjusted by family history of diabetes, hypertension, obesity, and dyslipidemia.

^b^Calculated in the additive, dominant or recessive model.

^c^Analyzed under the dominant model.

^d^Analyzed under the recessive model.

**Table 2:** Association between the serum levels of HCHs and the risk of T2DM.

Categorical levels of HCHs	Control No. (%)	Case No. (%)	OR (95% CI)^a^	*P*_*trend*_
HCHs				<0.001
Tertile 1	241 (59.2)	166 (40.8)	1.00 (Reference)	
Tertile 2	241 (55.8)	191 (44.2)	1.09 (0.76, 1.57)	
Tertile 3	241 (39.7)	366 (60.3)	2.31 (1.55, 3.44)	
α-HCH				0.142
<LOD	524 (48.6)	554 (51.4)	1.00 (Reference)	
Median 1^b^	99 (86.1)	16 (13.9)	0.18 (0.08, 0.36)	
Median 2^b^	100 (39.5)	153 (60.5)	1.24 (0.79, 1.94)	
β-HCH				<0.001
<LOD	333 (61.6)	208 (38.4)	1.00 (Reference)	
Median 1^b^	195 (46.8)	222 (53.2)	1.53 (1.09, 2.16)	
Median 2^b^	195 (40.0)	293 (60.0)	2.56 (1.67, 3.89)	
γ-HCH				0.307
<LOD	662 (51.6)	621 (48.4)	1.00 (Reference)	
Median 1^b^	31 (39.2)	48 (60.8)	1.43 (0.82, 2.53)	
Median 2^b^	30 (35.7)	54 (64.3)	1.43 (0.73, 2.81)	
δ-HCH				0.560
<LOD	604 (50.8)	585 (49.2)	1.00 (Reference)	
Median 1^b^	60 (53.1)	53 (46.9)	0.99 (0.57, 1.72)	
Median 2^b^	59 (41.0)	85 (59.0)	1.31 (0.79, 2.19)	

LOD: the limit of detection.

^a^Adjusted by family history of diabetes, hypertension, obesity, dyslipidemia and total lipids.

^b^Categorized by the detectable levels of controls.

**Table 3:** Interaction of *ADIPOQ* genotypes and β-HCH on the risk of T2DM.

Study model	Genotypes		β-HCH		Interaction	
	OR (95% CI)^a^	*P*^b^	OR (95% CI)^a^	*P*^b^	OR (95% CI)^a^	*P*^b^
rs182052 × β-HCH						
Additive model	0.86 (0.70, 1.06)	0.147	1.15 (1.05, 1.26)	0.003	2.20 (1.39, 3.49)	0.010
Recessive model	0.77 (0.53, 1.09)	0.144	1.21 (1.10, 1.31)	<0.001	2.13 (1.31, 3.26)	0.005
rs266729 × β-HCH						
Additive model	0.82 (0.67, 1.00)	0.055	1.19 (1.09, 1.30)	<0.001	1.07 (1.01, 1.15)	0.048
Recessive model	0.65 (0.45, 0.76)	0.066	1.25 (1.14, 1.36)	<0.001	1.04 (0.89, 1.22)	0.643
rs6810075 × β-HCH						
Additive model	0.99 (0.82, 1.22)	0.989	1.16 (1.06, 1.27)	0.002	1.09 (1.02, 1.17)	0.018
Recessive model	0.89 (0.62, 1.26)	0.501	1.21 (1.11, 1.31)	<0.001	1.14 (1.01, 1.28)	0.035
rs16861194 × β-HCH						
Additive model	0.99 (0.91, 1.08)	0.893	1.09 (0.84, 1.41)	0.517	0.99 (0.91, 1.08)	0.815
Recessive model	0.87 (0.40, 1.87)	0.720	1.25 (1.15, 1.36)	<0.001	1.22 (0.88, 1.71)	0.239

^a^Adjusted by family history of diabetes, hypertension, obesity, dyslipidemia, and total lipids.

^b^Bonferroni correction, *P < *0.0125 is considered as statistical significance.

**Table 4:** Combined effects of rs182052 and β-HCH on the plasma levels of adiponectin.

Categorical levels of β-HCH	GG		AG/AA		*P*^a^
	N	Median (Min, Max) (μg/mL)	N	Median (Min, Max) (μg/mL)	
<LOD	161	12.13 (0.62, 52.02)	380	10.67 (0.90, 62.02)	0.147
Median 1^b^	124	10.52 (0.31, 53.49)	293	10.25 (0.57, 48.03)	0.475
Median 2^b^	138	8.19 (0.50, 39.00)	350	8.04 (0.51, 53.86)	0.382
*P*^b^		0.173		0.016	

^a^Nonparametric test.

^b^Categorized by the median of detectable levels.

LOD: the limit of detection.
