# Supplementary material for: Interaction between β-hexachlorocyclohexane and ADIPOQ genotypes contributes to the risk of type 2 diabetes mellitus in East Chinese adults
Source: Sci Rep. 2016 Nov 24;6:37769. doi: 10.1038/srep37769 (PMC5121886; doi:10.1038/srep37769)
Supplement: Supplementary Information [file srep37769-s1.pdf]

Interaction between β-hexachlorocyclohexane and *ADIPOQ* genotypes contributes to the risk of type 2 diabetes mellitus in East Chinese adults

Shushu Li, Xichen Wang, Lu Yang, Shen Yao, Ruyang Zhang, Xue Xiao, Zhan Zhang, Li Wang, Qiujin Xu, Shou-Lin Wang

Supplementary Information

Methods

Examination and definition of physiological and biological indices

Body mass index. During the health examination, both body weight in light clothing and height in standing position of the participants were measured without shoes; the body mass index (BMI) was calculated as weight (kg) divided by height squared (m^2). Following the WHO Asian criteria, two different cutoff values were used: 24 ≤ BMI < 28 (overweight) and BMI ≥ 28 (obesity).

Blood pressure. Systolic and diastolic blood pressure were measured in the sitting position after 5-minute rest. Three blood pressure readings were obtained at 1-minute intervals and averaged for use in the analyses. Hypertension was defined as systolic pressure ≥ 140 mmHg or diastolic pressure ≥ 90 mmHg.

Cigarette smoking and alcohol drinking. Cigarette smoking was defined as having smoked more than 1 cigarette every day for more than one year, and alcohol drinking was defined as alcohol intake more than three times per week during the past 6 months.

Biochemical indices. Serum biochemical indices were measured in community hospitals, including total cholesterol (TC), triglycerides (TG), high-density lipoprotein cholesterol (HDLC), low-density lipoprotein cholesterol (LDLC), alanine transaminase (ALT), aspartate aminotransferase (AST), blood urea nitrogen (BUN), and blood uric acid. Each of the biochemical indices was evaluated according to the "Prevention and treatment of dyslipidemia in Chinese adults (2007) [1]". Hyperuricemia was defined as a concentration of blood uric acid > 7 mg/dL in males and > 6 mg/dl in females. Hypercholesterolemia was defined as TC ≥ 6.22 mmol/L, and hypertriglyceridemia was defined as TG ≥ 2.26 mmol/L. The definition of dyslipidemia required that the participant satisfied two or more of the following four

criteria: 1) TC ≥ 6.22 mmol/L; 2) TG ≥ 2.26 mmol/L; 3) HDLC < 1.4 mmol/L; and 4) LDLC ≥ 4.14 mmol/L.

Analysis of hexachlorocyclohexanes (HCHs) in serum

Extraction of serum samples. The extraction, separation and cleanup methods employed in the study were modified from a previous study [2]. Briefly, 1 mL serum sample was denatured with hydrochloric acid (HCl) and 2-propanol, then extracted with methyl tert-butyl ether (MTBE):n-hexane (1:1, v/v) three times. The eluent was dried by nitrogen sweeping and dissolved in 1 mL dichloromethane (DCM) : n-hexane (1:1, v/v). Subsequently, a solid phase extraction (SPE) column (500 mg, 6 mL, CNW) was activated with n-hexane. After conditioning, 1 mL solution was added into the wet SPE column. Subsequently, 6 mL dichloromethane (DCM) : n-hexane (1:1, v/v) was added to the column for elution. Finally, the extract was concentrated to a volume of 0.1 mL. All glass tubes were treated with potassium permanganate and potassium dichromate sulfuric acid. All solvents underwent pesticide residue analysis quality, and the reagent water for aqueous solutions was Optima quality.

Analysis of HCHs. A gas chromatograph-tandem mass spectrometer (GC-MS/MS, TSQ8000, Thermo Fisher Scientific, USA) was used to detect the levels of HCHs in human serum. A DB-5u (30 m × 0.25 mm inner diameter × 0.25 mm film thickness; J&W Scientific, Folsom, CA) capillary column was used with helium as the carrier gas at 1.0 mL/min. The initial temperature was 100°C for 2.5 min, then increased to 150 for 2.5 min at 20°C/min, and then increased to 310°C for 5 min at 40℃/min. The injector and source temperatures were set at 275℃ and 220℃, respectively. A 1 μL treated reconstituted sample was injected using the splitless injection mode for 1 min. The mass spectrometer was operated under electron ionization. Auto SRM was employed to determine individual peaks.

Quality Control of analysis. The samples were analyzed randomly within a larger project with 100 total batches of samples. For a batch analysis of 15 serum samples, one procedure blank consisting of purified water, and one in-house reference standard consisting of pooled human serum were analyzed. Hexane was used for flushing needles or columns, and quality control mixture standards were analyzed for every 15 samples analyzed. The analytic results were reported on wet-weight basis, and the serum lipid levels were included as a covariate in the regression analysis. Total lipids in plasma were calculated using the formula of Bernert et al. [3]:

$$\text{Total lipids} = (2.27 \times \text{total cholesterol}) + \text{triglycerides} + 62.3\ \text{mg/dL}$$

The limit of detection (LOD) was defined as three times signal to noise ratio, and samples below LOD were given half the value of each LOD value[4]. The reproducibility of the method was demonstrated by 12 replicate determinations using an in-house control serum sample among the analytical batches during the course of the study. The average recoveries of the different congeners added to serum samples were 111 ± 3%, which showed that the loss of HCHs was negligible during the analytical process.

Results

A total of 250 random serum samples were evaluated in the preliminary screening for the detection of all 4 HCH isomers. The β-HCH ranked first, with a detection rate of 80.26%; other isomers, including α-HCH, γ-HCH and δ-HCH, had detection rates of 6.14%, 33.77%, 23.42%, respectively. Finally, 4 HCH isomers were detected in 1446 serum samples. The results suggest that the detection rates of α-HCH, β-HCH, γ-HCH and δ-HCH were 25.5%, 62.5%, 11.0% and 8.0%, respectively, among which β-HCH presented the highest detection rate and concentration. In addition, both the detection rate and the geometric mean concentration of β-HCH in

serum were much higher in cases than in controls (Table S6). The recovery, relative standard deviation (RSD), and limits of detection (LOD) of β-HCH in human serum are listed in Table S4. As shown in Fig. S2, the bottom of the peak was low and relatively clean, the treatment of material interference was small, and the method of detection should be considered reasonable.

References

1 Joint Committee for Developing Chinese guidelines, on Prevention Treatment of Dyslipidemia in & Adults. Chinese guidelines on prevention and treatment of dyslipidemia in adults. *Chin J Cardiol* **35**, 390-419 (2007). Chinese

2 Bradman, A. *et al.* Polybrominated diphenyl ether levels in the blood of pregnant women living in an agricultural community in California. *Environ Health Perspect* **115**, 71-74 (2007).

3 Bernert, J. T., Turner, W. E., Patterson, D. G., Jr. & Needham, L. L. Calculation of serum "total lipid" concentrations for the adjustment of persistent organohalogen toxicant measurements in human samples. *Chemosphere* **68**, 824-831 (2007).

4 Ling, H. *et al.* Genome-wide linkage and association analyses to identify genes influencing adiponectin levels: the GEMS Study. *Obesity (Silver Spring)* **17**, 737-744 (2009).

Table S1 Distribution of main characteristics in cases and controls

Variables	Control (N=723)	Case (N=723)	*P*
	n (%)	n (%)	
Age (years old)	61.58±11.58	61.59±11.58	0.984
Sex			1.000
male	241 (33.3%)	241 (33.3%)	
female	482 (66.7%)	482 (66.7%)	
Cigarette smoking	171 (23.7)	170 (23.5)	0.951
Alcohol drinking	117 (16.2)	128 (17.7)	0.441
Family history of diseases			
hypertension	81 (11.2)	86 (11.9)	0.681
hyperlipidemia	8 (1.1)	9 (1.2)	0.807
diabetes	16 (2.2)	43 (5.9)	< 0.001
Hypertension	247 (34.2)	454 (62.8)	< 0.001
Obesity	51 (7.1)	171 (23.7)	< 0.001
Dyslipidemia	112 (15.5)	363 (50.2)	< 0.001
Biochemical indices			< 0.001
TC ≥ 6.22 mmol/L	22 (3.0)	124 (17.2)	< 0.001
TG ≥ 2.66 mmol/L	22 (3.0)	209 (28.9)	< 0.001
HDLC < 1.40 mmol/L	85 (11.8)	185 (25.6)	< 0.001
LDLC ≥ 4.14 mmol/L	17 (2.4)	96 (13.3)	< 0.001
ALT > 40 U/L	9 (1.2)	9 (1.2)	1.000
AST > 40 U/L	36 (5.0)	32 (4.4)	0.619
BUN > 7.14 mmol/L	144 (19.9)	168 (23.2)	0.125
Hyperuricemia	37 (5.1)	45 (6.2)	0.363

Table S2 Independent risk factors of T2DM by conditional logistic regression analysis

Risk factors	*B*	*Wald*	OR (95% CI)	*P*
Family history of diabetes	1.05	7.85	2.87 (1.37, 5.99)	0.005
Hypertension	1.22	57.20	3.38 (2.47, 4.64)	< 0.001
Obesity	1.09	24.63	2.99 (1.94, 4.61)	< 0.001
Dyslipidemia	1.89	101.03	6.61 (4.58, 9.56)	< 0.001

Table S3 Primers and probe sequences for the amplification of *ADIPOQ* genotypes

NCBI rs #	Primer Sequence (5'-3')	Probe Sequence (5'-3')
rs182052	F:CCTTCTAAGATGCATGGAACCAT	G:FAM-TGAATTTTGCCCAGTTC-MGB
G>A	R:ACTTTTTCTCAATAGGTATCCTGCTACA	A:HEX-CTGAATTTTACCCAGTTCG-MGB
rs266729	F:CAGAATGTGTGGCTTGCAAGA	C:FAM-TCAGATCCTGCGCTT-MGB
C>G	R:TGGCACGCTCATGTTTTGTT	G:HEX-AGATCCTGCCCTTCA-MGB
rs6810075	F:GACATGTCTACCAGGTCCAAAAAA	T:FAM-TCCTACACAATCTGCGG-MGB
T>C	R:AAGACGGAATGCATAGGTGTGTATT	C:HEX-TCCTACACAATCCGCGGT-MGB
rs16861194	F:TCTGAGCCGGTTCTTGCAA	A:FAM-ATGAATTAAATTACGACCCC-MGB
A>G	R:TGGTGCTGGCATCCTAAGC	G:HEX-CATTCTGATGAATTAAACTACGA-MGB

Table S4 Basic characteristics of *HCH* isomers standards in GC-MS/MS analysis

Compound	Retention time (min)	Linear range (ng/mL)	qualifier/quantitative ion (m/z)	r	LOD (ng/mL)	RSD (%)	Recovery Rate (%)
α-HCH	10.92	1-50	35,71*,255	0.9983	0.02	1.40	110.40
β-HCH	11.33	1-50	35,71*,255	0.9999	0.08	2.70	115.40
γ-HCH	11.40	1-50	35,71*,255	0.9987	0.03	2.60	116.70
δ-HCH	11.73	1-50	35,71,255*	0.9987	0.09	1.00	115.10

LOD: the limit of detection; RSD, relative standard deviation; * presented quantitative ion.

Table S5 Basic characteristics and MAF of *ADIPOQ* SNPs in the study

SNP	CHR	Allele	MAF-CHB	MAF-case	MAF-control	*P*-HWE
rs182052	3	G>A	0.41	0.47	0.47	0.49
rs266729	3	C>G	0.30	0.28	0.30	0.15
rs6810075	3	T>C	0.41	0.47	0.46	0.71
rs16861194	3	A>G	0.17	0.19	0.18	0.27

[a] CHR, Chromosome; MAF, Minor allele frequency; CHB, China-Han-Beijing; HWE, Hardy-Weinberg equilibrium

Table S6 Comparison of the serum levels of *HCH* isomers in cases and controls

HCH isomers	Case	Control	*P*
**α -HCH**			
Detection Rate (%)	23.4	27.5	0.070
Geometric mean (95% CI) (ng/mL) [a]	0.012 (0.012, 0.013)	0.011 (0.011, 0.012)	0.199
**β -HCH**			
Detection Rate (%)	71.2	53.9	0.000
Geometric mean (95% CI) (ng/mL) [a]	0.575 (0.500, 0.661)	0.266 (0.233, 0.303)	0.000
**γ -HCH**			
Detection Rate (%)	14.1	8.4	0.001
Geometric mean (95% CI) (ng/mL) [a]	0.020 (0.019, 0.022)	0.018 (0.017, 0.019)	0.595
**δ -HCH**			
Detection Rate (%)	19.1	16.5	0.191
Geometric mean (95% CI) (ng/mL) [a]	0.068 (0.063, 0.073)	0.060 (0.057, 0.064)	0.636

[a] Data below the limit of detection (LOD) is given a half value of LOD.

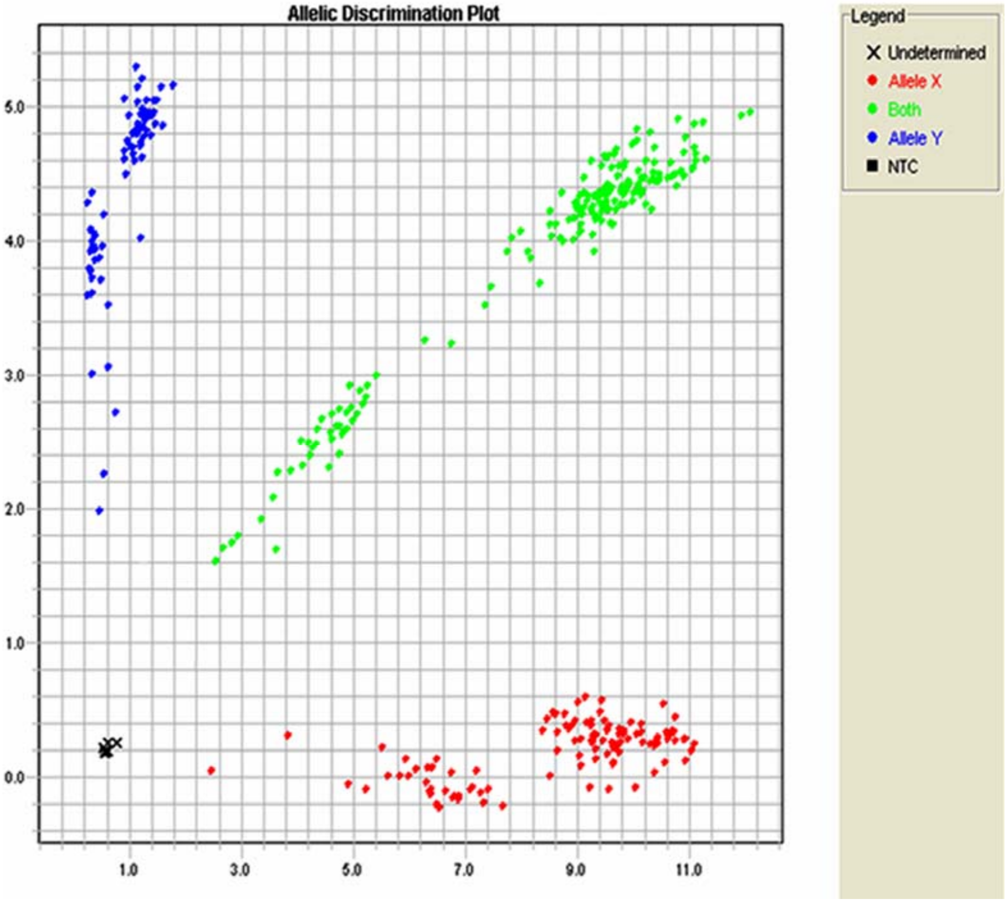

Fig. S1 ***ADIPOQ*** **genotype by TaqMan analysis**. Blue scatters, wild homozygote; green scatters, heterozygote; red scatters, mutant homozygote; black scatters, blank correction.

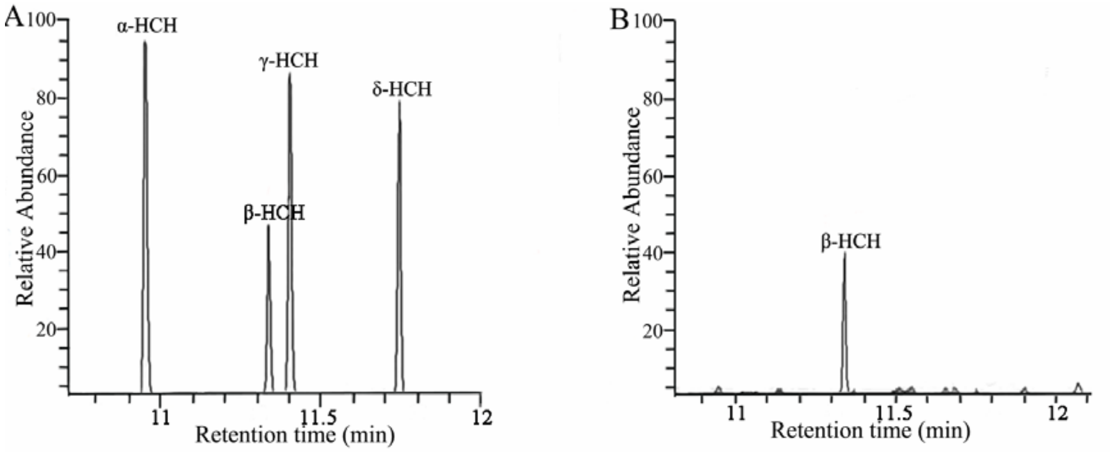

Fig. S2 Spectrum of HCH isomers for standards or serum sample. The samples were detected using GC-MS/MS. **(A)** Spectrum of the HCH standards mixture (100 ng/mL). **(B)** Spectrum of β-HCH in human serum; 1 μl serum sample was used.
